# Philanthropy for global mental health 2000–2015

**DOI:** 10.1017/gmh.2020.2

**Published:** 2020-05-06

**Authors:** Valentina Iemmi

**Affiliations:** 1 Department of Social Policy, London School of Economics and Political Science, London, UK; 2 Department of Health Policy, London School of Economics and Political Science, London, UK

**Keywords:** Development assistance for health, low- and middle-income countries, global mental health, philanthropy, sustainable financing

## Abstract

**Background:**

Mental disorders are the leading cause of years lived with disability worldwide. While over three-quarters of people with mental disorders live in low- and middle-income countries (LMICs) and effective low-cost interventions are available, resource commitments are extremely limited. This paper seeks to understand the role of philanthropy in this area and to inform discussions about how to increase investments.

**Methods:**

Novel analyses of a dataset on development assistance for health were conducted to study philanthropic development assistance for mental health (DAMH) in 156 countries between 2000 and 2015.

**Results:**

Philanthropic contributions more than doubled over 16 years, accounting for one-third (US$364.1 million) of total DAMH 2000–2015. However, across health conditions, mental disorders received the lowest amount of philanthropic development assistance for health (0.5%). Thirty-seven of 156 LMICs received no philanthropic DAMH between 2000 and 2015 and just three LMICs (Antigua and Barbuda, Grenada, Saint Vincent and the Grenadines) received more than US$1 philanthropic DAMH per capita over the entire period. Eighty-one percent of philanthropic DAMH was disbursed to unspecified locations.

**Conclusions:**

Philanthropic donors are potentially playing a critical role in DAMH, and the paper identifies challenges and opportunities for increasing their impact in sustainable financing for mental health.

## Introduction

Mental disorders (including substance use disorders, dementia and self-harm) are the leading cause of years lived with disability worldwide (19%) (Global Burden of Disease Collaborative Network, 2018*b*; IHME, 2018*c*). While over three-quarters of people with mental disorders live in low- and middle-income countries (LMICs) fewer than 10% receive treatment (WHO, 2018*b*). Investments in mental disorders in LMICs are extremely limited: only 1.6% of LMIC government health budgets (WHO, 2018*b*) and 0.4% of development assistance for health (DAH, i.e. financial and in-kind contributions for health disbursed by donors to LMICs) (Charlson *et al*., 2017). With LMIC government budgets often at capacity, it is paramount to mobilise additional external resources (Patel *et al*., 2018).

United Nations Sustainable Development Goals recommend external resources for development from a wide range of sources, including philanthropy (UN, 2015*b*). Philanthropy includes contributions from non-state actors such as foundations, corporations and individuals (Youde, 2018). Over the last two decades their role and influence in global health has increased, bringing additional resources and innovative ideas along with concerns about legitimacy (Youde, 2018) and conflicts of interest (Stuckler *et al*., 2011). While philanthropic contributions account for 17% of DAH (Dieleman *et al*., 2016), they represent over one-third of development assistance for mental health (DAMH) (Charlson *et al*., 2017). This paper analyses philanthropic DAMH in 156 countries between 2000 and 2015 to understand the role of philanthropy in this area and inform discussions about how to increase investments to address mental disorders.

## Methods

I merged the Institute of Health Metrics and Evaluation (IHME) dataset on DAH 1990–2017 (IHME, 2018*a*) with three variables: country classification per region (WHO, 2018*a*), per country income-level (World Bank, 2018) and country population size (Global Burden of Disease Collaborative Network, 2018*a*). The IHME DAH dataset reports estimates on primary sources of funding for 172 countries (1990–2017), 24 governments and philanthropic donors (corporations, foundations, individuals) (IHME, 2018*b*). Estimates are also provided on *channels*, defined as intermediary organisations disbursing funding to implementing institutions providing support in LMICs. These channels include bilateral governmental organisations (e.g. United Kingdom Department for International Development), multilateral governmental organisations (e.g. World Health Organization, WHO), multilateral development finance institutions (e.g. World Bank), non-governmental organisations, United States (US) foundations and global health initiatives (e.g. Global Fund to Fight AIDS, Tuberculosis and Malaria). US foundations can be either primary sources or channels. Recipient countries are classified as unspecified by IHME when information is not available.

I conducted descriptive analyses of philanthropic DAMH by year in absolute and relative terms, and compared with philanthropic DAH to other health conditions (HIV/AIDS, tuberculosis, malaria, other infectious diseases, maternal health, newborn and child health, non-communicable diseases excluding mental health), by channel organisation, and by recipient country. I limited analyses to 2000–2015, due to poor data quality pre-2000, preliminary estimates post-2015 and focus on the Millennium Development Goals era to inform the Sustainable Development Goals era, leaving 168 countries. I excluded 12 small overseas territories or dependencies due to the lack of World Bank country classification. Among excluded countries, only two received non-philanthropic DAMH during the period, Anguilla (2005) and the Cook Islands (2005–2006 and 2008–2012). Values are reported in 2017 United States dollars (US$) adjusted by purchasing-power parity. Analyses were conducted in Stata 14. Online Supplementary Appendix 1 provides further details.

## Results

### Annual trends

**Fig. 1.**
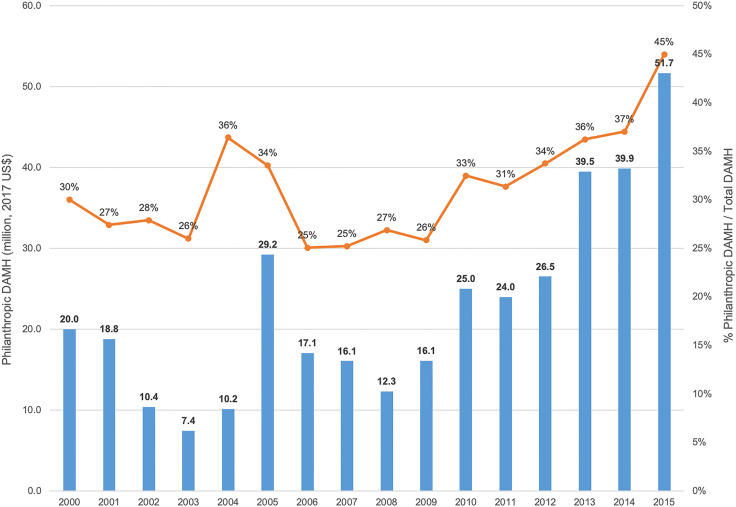
Annual philanthropic DAMH as a percentage of total DAMH between 2000 and 2015 (million, 2017 US$). DAMH, development assistance for mental health.

Between 2000 and 2015, philanthropic DAMH amounted to US$364.1 million, representing one-third of total DAMH (online Supplementary Appendix 2). Philanthropic contributions within DAMH increased substantially, both in absolute terms (more than doubling from US$20 million to US$51.7 million) and in relative terms (30% to 45% of total DAMH; Fig. 1). By contrast, over the same period, philanthropic DAH represented a smaller (17%) and constant share of DAH (online Supplementary Appendix 2).

**Fig. 2.**
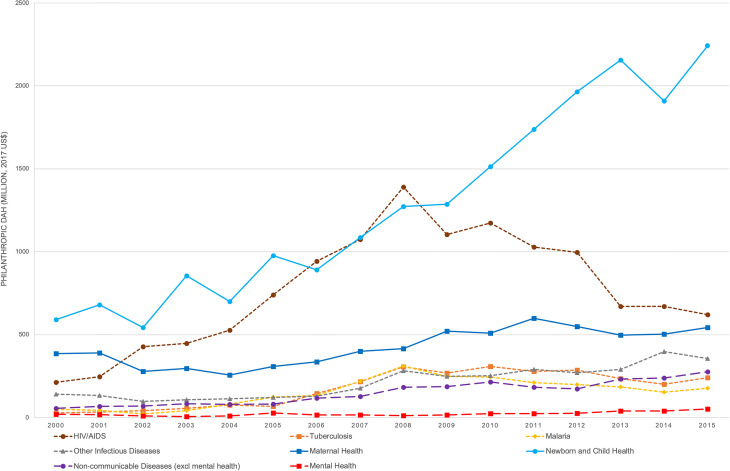
Annual philanthropic DAH across health conditions between 2000 and 2015 (million, 2017 US$). DAH, development assistance for health.

Over 16 years, mental disorders received the lowest amount (0.5%) of philanthropic DAH across health conditions (online Supplementary Appendix 3). Newborn and child health (28%) and HIV/AIDS (17%) received the largest amounts. Over 16 years, philanthropic DAMH increased 2.6-fold (US$20 million to US$52 million), slightly lower than the 3.3-fold increase in philanthropic DAH (Fig. 2). While philanthropic DAH experienced substantial changes over the period for some health conditions (e.g. newborn and child health, HIV/AIDS), the increase was less sizeable for mental disorders.

### Channel organisations

Between 2000 and 2015, non-governmental organisations were the main channels of philanthropic DAMH (US$254 million), followed by US foundations (US$79 million) and multilateral governmental organisations (US$31 million) (online Supplementary Appendix 4). Over 16 years, the proportion of philanthropic DAMH doubled for non-governmental organisations (38% to 77%) but more than halved for foundations (32% to 14%) and reduced even more noticeably for multilateral governmental organisations (30% to 9%). Non-governmental organisations were the main channels of philanthropic DAH (US$39 334 million) followed by US foundations (US$20 357 million), multilateral governmental organisations (US$8901 million) and global health initiatives (US$3847 million). Relative shares remained stable over the period.

**Fig. 3.**
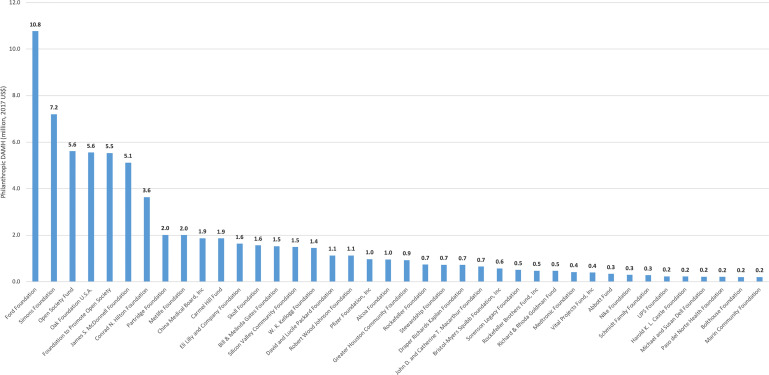
Cumulative philanthropic DAMH by the top 40 US foundations as channels between 2000 and 2015 (million, 2017 US$). DAMH, development assistance for mental health.

Among US foundations, Ford Foundation (US$11 million) was the largest channel for philanthropic DAMH over the period, followed by Simons Foundation (US$7 million) and Open Society Fund and Oak Foundation (US$6 million each) (Fig. 3). There were variations in the most generous US foundations channelling funding across regions and country-income groups (online Supplementary Appendix 5). Across regions, Ford Foundation was the largest contributor in four regions (Africa, Eastern Mediterranean, South-East Asia and Western Pacific), Open Society Fund in Europe and James S. McDonnel Foundation in the Americas. Similarly, Ford Foundation was the largest contributor in low-income (US$1.7 million) and lower middle-income countries (US$5.2 million), while James S. McDonnel Foundation was the largest in upper middle-income countries (US$2.9 million).

### Recipient countries

The majority of philanthropic DAMH between 2000 and 2015 was disbursed to unspecified locations (81%) or multiple regions (10%) (online Supplementary Appendix 6). Amongst known recipient countries, philanthropic DAMH varied across regions and country-income groups. It accounted for more than one-third of DAMH to both Western Pacific (US$11 million) and the Americas (US$12 million) unlike less than 5% to Eastern Mediterranean (US$3 million) and Africa (US$4 million). It represented over one-quarter of DAMH to upper middle-income countries (US$14 million) but 5% to low-income countries (US$6 million).

**Fig. 4.**
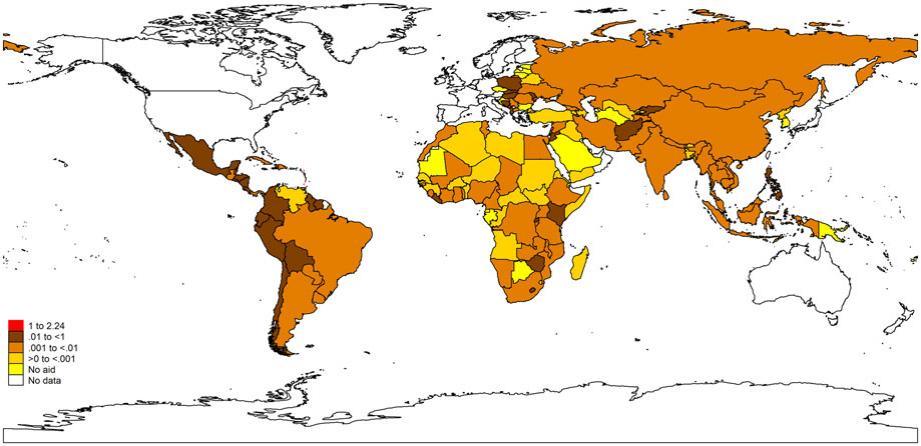
Cumulative philanthropic DAMH per capita in recipient countries between 2000 and 2015 (2017 US$). DAMH, development assistance for mental health.

Across known recipient countries, philanthropic DAMH varied broadly. Over 16 years, China was the largest recipient (US$6 million), followed by the Philippines (US$4 million), Mexico (US$3 million) and Brazil (US$2 million). However, considering per capita estimates, only three out of 156 LMICs received more than US$1 per capita over the entire period (Antigua and Barbuda, Grenada, Saint Vincent and the Grenadines) (Fig. 4). Thirty-seven countries received no philanthropic DAMH: nine African, four American, two Eastern Mediterranean, seven Eastern European, one South-East Asian and 11 Western Pacific countries (online Supplementary Appendix 6).

## Discussion

The paper offers a detailed account of trends in philanthropic DAMH in 156 countries between 2000 and 2015. Philanthropic contributions represented one-third (US$364.1) of total DAMH, more than doubling over 16 years. However, across health conditions, mental disorders received the lowest amount of philanthropic DAH (0.5%). Philanthropic DAMH was mainly channelled through non-governmental organisations (US$254 million). More than one-third of DAMH to Western Pacific and the Americas was philanthropic. The analyses suggest philanthropic contributions to mental disorders represented a small share of philanthropic DAH but had a substantial and increasing role in DAMH.

These results highlight four main challenges for philanthropic DAMH: scarcity, sustainability, allocation and data. Philanthropic contributions to mental disorders were limited, accounting for a relatively small share of philanthropic DAH when compared to other health conditions, reflecting similar trends in high-income countries (Brousseau *et al*., 2003). The substantial share of DAMH disbursed by philanthropy raises concerns regarding its sustainability, especially vis-à-vis volatility and fungibility (i.e. partial displacement of domestic health budgets). While volatility concerns reflect broader challenges in DAH (Moon and Omole, 2017), philanthropy accounted for a lower share (less than 10%) of DAH across regions and country-income groups. Fungibility of philanthropic DAMH is partly mitigated by large disbursements through non-governmental organisations, which have been shown to have positive impacts on domestic government health spending (Lu *et al*., 2010).

The uneven allocation of philanthropic DAMH means that the region where the majority of people with mental disorders live, South East Asia (26%) (Global Burden of Disease Collaborative Network, 2018*b*), received only 17% of philanthropic DAMH, raising concerns about equitable allocation. A similar misalignment occurs with total DAMH (Gilbert *et al*., 2015; Charlson *et al*., 2017) and DAH (Dieleman *et al*., 2014). While allocation of development assistance is determined by a variety of factors beyond needs, including policy environment and donor interests (Hoeffler and Outram, 2011), stakeholders recognise health needs as of primary concern (Ottersen *et al*., 2018). Similarly, factors beyond needs drive philanthropic giving, including solicitation, cost-benefit, altruism, reputation, psychological benefits, values and efficacy (Bekkers and Wiepking, 2011).

Finally, data on philanthropic DAMH are extremely poor in coverage and quality. They focus predominantly on US foundations and they are often insufficiently disaggregated. For instance, organisation names at the source and channel level are available only for Bill & Melinda Gates Foundation (BMGF) and US foundations, respectively. BMGF disbursing 15% of DAMH only as a channel (Charlson *et al*., 2017) suggests other US foundations could disburse potentially a much larger amount through other channels. This reflects the lack of transparency of philanthropic donors in development (OECD, 2018).

This analysis has limitations due to data constraints. First, data are limited in breadth, focusing predominantly on US foundations. While this may have excluded some key players, almost three-quarters of philanthropic contributions in development originate from the US (OECD, 2018). Second, data are limited in depth, so that estimates are conservative for *some* organisations. For instance, IHME classifies DAH channelled through global health initiatives and some multilateral governmental organisations (United Nations Children's Fund, Joint United Nations Programme on HIV and AIDS) to health conditions constituting the organisations' focus, although programmes may include mental health components (IHME, 2018*b*).

Third, data are limited in scope, focusing on health only. This may have excluded sectors directly or indirectly relevant to mental health (e.g. education, employment) (Lund *et al*., 2018). Fourth, data are limited in granularity. For instance, the majority of contributions are disbursed to unspecified countries and no information is reported on activities funded and populations targeted, limiting interpretations. Finally, inclusion of some neurological conditions (epilepsy, headache disorders, Parkinson's disease) reflects prior conceptualisation of mental disorders (WHO, 2008) and may have increased estimates.

The analyses in this paper show that, among external actors (Iemmi, 2019), philanthropic donors are already playing critical, albeit limited and imperfect, roles in DAMH. I suggest four opportunities for maximising their impact. First, philanthropic donors could initiate or increase contributions to mental disorders to reflect their growing relative importance as part of the epidemiological transition in LMICs (GBD 2017 DALYs and HALE Collaborators, 2018). They could scale-up their efforts through their priorities and competitive advantages, as illustrated for 15 large international foundations by the *Lancet Commission on Global Mental Health and Sustainable Development* (Patel *et al*., 2018, online Supplementary Table S5).

Second, in line with Sustainable Development Goals and Addis Ababa Action Agenda (UN, 2015*a*), they could adopt a sustainable approach to disbursements in order to assure local ownership and impact beyond funded activities. They could *systematically* encourage partnerships between implementing organisations and local actors to facilitate an incremental transition to domestic delivery and funding (WHO, 2013). Third, philanthropic DAMH could be allocated within organisations' strategic roles and priorities, but more equitably across countries, reflecting local needs (e.g. burden of mental disorders) (Global Burden of Disease Collaborative Network, 2018*b*), capacity (e.g. mental health system) (WHO, 2018*b*) and recommended interventions and approaches (Patel *et al*., 2016). Finally, philanthropic donors could increase transparency, collecting and sharing better and more disaggregated data. This could inform the work of organisations tracking resources (OECD, 2017; IHME, 2018*a*) and monitoring global efforts in mental health (Saxena *et al*., 2019), paramount for informing funding decision and ultimately for sustainable financing for global mental health. Additional external resources for global mental health are urgently needed: philanthropy is a crucial actor and could amplify its impact embracing greater sustainability, better allocation and transparency.

## Supplementary material

For supplementary material accompanying this paper visit http://dx.doi.org/10.1017/gmh.2020.2. click here to view supplementary material
