# Supplementary material for: Philanthropy for global mental health 2000–2015
Source: Glob Ment Health (Camb). 2020 May 6;7:e9. doi: 10.1017/gmh.2020.2 (PMC7379329; doi:10.1017/gmh.2020.2)
Supplement: Supplementary file 1 [file S2054425120000023sup001.pdf]

Supplementary appendix

Supplement to: Iemmi V (2020). Philanthropy for global mental health 2000–2015. Global Mental Health 1–6. https://doi.org/10.1017/gmh.2020.2

Supplementary appendix

Appendix 1. Data sources and analyses……………………………………………………….…..2

Appendix 2. Annual philanthropic DAMH and annual philanthropic DAH between 2000 and 2015 (million, 2017 US$) and as proportion of annual total DAMH and DAH...……………..…...…..5

Appendix 3. Annual philanthropic DAH for mental health and other health conditions between 2000 and 2015 (million, 2017 US$)…………………………………………………………..…...6

Appendix 4. Annual philanthropic DAMH and annual philanthropic DAH between 2000 and 2015, by channel organisation (million, 2017 US$)…………………………….…………….…..…..7

Appendix 5. Cumulative philanthropic DAMH by the top 10 US foundations as channels between 2000 and 2015, by region and country income group (thousand, 2017 US$)………..8

Appendix 6. Cumulative philanthropic DAMH and philanthropic DAH between 2000 and 2015, by region, country income group, country (thousand, 2017 US$)……………………….………11

Appendix 1. Data sources and analyses

Data sources

I merged the Institute of Health Metrics and Evaluation (IHME) dataset on development assistance for health (DAH) 1990-2017 (IHME, 2018) with three variables: country classification per region (WHO, 2018), per country income-level (World Bank, 2018), and country population size (Global Burden of Disease Collaborative Network, 2018). DAH includes "in-kind and financial resources transferred from primary development channels to low-income and middle-income countries for the purpose of maintaining or improving health" (Dieleman *et al.*, 2016, p. 2537).

The IHME DAH dataset reports semi-aggregated data on DAH in 172 countries between 1990 and 2017 (IHME, 2018). It reports estimates on resource flows from funding sources (Table 1), through *channel* organisations, defined as intermediary organisations disbursing funding to implementing institutions providing support in low- and middle-income countries (Table 2). The dataset is built by IHME using different sources: Development Assistance Committee and Creditor Reporting System databases (Organisation for Economic Co-operation and Development), financial reports, audited financial statements, United States Agency for International Development Report of Voluntary Agencies, Foundation Center's grant database, Bill & Melinda Gates Foundation online grant database, Internal Revenue Service 990 tax forms, and personal correspondences (Global Burden of Disease Health Financing Collaborator Network, 2018).

These data, in an aggregated form, exists publically on the Global Health Data Exchange (IHME, 2018). A detailed dataset was obtained from IHME in September 2018, including values omitted in the publicly available dataset (i.e. values greater than US$0 but less than US$500, or less than US$0 and greater than -US$500). In addition, disaggregated data for United States foundations (variable *channel*, category *Other US Foundations* in Table 2) were obtained in June 2018.

It is worth noting that development assistance for mental health in the IHME DAH dataset captures not only mental disorders (including substance use disorders, dementia, and self-harm) but also some neurological conditions (epilepsy, headache disorders, Parkinson's disease). This reflects previous conceptualisations of mental disorders (WHO, 2008). At the time of the analyses for this paper it was not possible to access data on development assistance for mental health excluding those neurological conditions.

Table 1. *Funding sources*

Donors	Description
Governments (OECD DAC members)	Australia; Austria; Belgium; Canada; Denmark; Finland; France; Germany; Greece; Ireland; Italy; Japan; Korea; Luxembourg; Netherlands; New Zealand; Norway; Portugal; Spain; Sweden; Switzerland; United Kingdom; United States
Governments (non OECD DAC members)	United Arab Emirates
**Bill & Melinda Gates Foundation**	Contribution from the Bill & Melinda Gates Foundation to NGOs

Donors	Description
**Corporate donations**	Private sector in-kind contributions to NGOs
**Private (other)**	Private sector financial contributions, including corporations, foundations (within and outside the United States), individuals, etc.
Debt repayments	Debt repayments (World Bank; regional development banks)
Other	Interest, transfer of funds, refunds, miscellaneous income earned by channel
Unallocable	Unspecified donor sector

Adapted from the dataset user guide (IHME, 2018). NGOs, non-governmental organisations; OECD DAC, Organisation for Economic Co-operation and Development's Development Assistance Committee. Bold: philanthropic donors included in the analyses.

Table 2. *Channel organisations*

Channel type	Organisations
Bilateral governmental organisations	Australia; Austria; Belgium; Canada; Denmark; Finland; France; Germany; Greece; Ireland; Italy; Japan; Korea; Luxembourg; Netherlands; New Zealand; Norway; Portugal; Spain; Sweden; Switzerland; United Arab Emirates; United Kingdom; United States
Multilateral organisations	European Commission; Pan-American Health Organization; Joint United Nations Programme on HIV/AIDS; United Nations Population Fund; United Nations Children's Fund; World Health Organization
Multilateral Development Finance Institutions	African Development Bank; Asian Development Bank; Inter-American Development Bank; World Bank, International Bank for Reconstruction and Development; World Bank, International Development Association
**Foundations**	Bill & Melinda Gates Foundation; Other United States Foundations
Non-Governmental Organisations	Sample of United States-based and internationally based non-governmental organisations receiving support from the United States government
Global Health Initiatives	Gavi, the Vaccine Alliance; Global Fund to Fight AIDS, Tuberculosis, and Malaria; Unitaid

Adapted from the dataset user guide (IHME, 2018). Bold: philanthropic donors included in the additional disaggregated dataset.

Analyses

I conducted descriptive analyses of annual philanthropic development assistance for mental health (DAMH) in absolute and relative terms, by channel organisation, by recipient country, and compared with philanthropic DAH to other health conditions (HIV/AIDS, tuberculosis, malaria, other infectious diseases, maternal health, newborn and child health, non-communicable diseases excluding mental health). Philanthropic donors included in the analyses are corporations, foundations, individuals (Table 1-2). Analyses were limited to 2000-2015, due to poor data quality pre-2000, preliminary estimates post-2015 and to focus on the Millennium Development Goals era to inform the Sustainable Development Goals (SDGs) era, leaving 168 countries.

I excluded 12 small overseas territories or dependencies due to lack of World Bank country classification: Anguilla, Cook Islands, Mayotte, Montserrat, Nauru, Niue, Saint Helena, Saint Martin, Tokelau, Turks and Caicos Islands, Tuvalu, Wallis and Futuna Islands. None of them received philanthropic DAMH. Only two countries received non-philanthropic DAMH during the period, Anguilla (2005) and the Cook Islands (2005-2006 and 2008-2012). To reflect disbursements to recipient countries dissolved or created during the period of study (Kosovo, Serbia, South Sudan), the World Bank country classification was imputed using the first observation carried backward and the last observation carried forward.

Transfers between channels captured elsewhere in the database were excluded to avoid double-counting. Values are reported in 2017 United States dollars (US$) adjusted by purchasing-power parity. Analyses were conducted in Stata 14.

References

Dieleman JL, Schneider MT, Haakenstad A, Singh L, Sadat N, Birger M, Reynolds A, Templin T, Hamavid H, Chapin A, Murray CJ (2016). Development assistance for health: past trends, associations, and the future of international financial flows for health. *The Lancet* **387**, 2536–2544.

Global Burden of Disease Collaborative Network (2018a). Global Burden of Disease Study 2017 (GBD 2017) Population Estimates 1950-2017. Seattle: Institute for Health Metrics and Evaluation. (http://ghdx.healthdata.org/record/global-burden-disease-study-2017-gbd-2017-population-estimates-1950-2017). Accessed 27 August 2019.

Global Burden of Disease Health Financing Collaborator Network 2018. Spending on health and HIV/AIDS: domestic health spending and development assistance in 188 countries, 1995-2015. *The Lancet* **391**, 1799–1829.

IHME (2018a). Development Assistance for Health Database 1990-2017. Seattle: Institute for Health Metrics and Evaluation. (http://ghdx.healthdata.org/record/ihme-data/development-assistance-health-database-1990-2017). Accessed 27 August 2019.

WHO (2008). mhGAP Mental Health Gap Action Programme. Scaling Up Care for Mental, Neurological, and Substance Use Disorders. Geneva: World Health Organization.

WHO (2018a). Global Health Observatory (GHO) Data. Geneva: World Health Organization. (https://www.who.int/gho/database/en/). Accessed 27 August 2019.

World Bank (2018). World Bank Country and Lending Groups (June 2018 edition). Washington DC: The World Bank. (https://datahelpdesk.worldbank.org/knowledgebase/articles/906519-world-bank-country-and-lending-groups). Accessed 27 August 2019.

Appendix 2. Annual philanthropic DAMH and annual philanthropic DAH between 2000 and 2015 (million, 2017 US$) and as proportion of annual total DAMH and DAH

	Philanthropic DAMH		Philanthropic DAH	
	US$ (million)	% Total DAMH	US$ (million)	% Total DAH
**2000**	20.0	29.5%	2,053.6	17.1%
**2001**	18.8	27.4%	2,232.9	17.8%
**2002**	10.4	27.9%	2,148.7	15.1%
**2003**	7.4	26.0%	2,588.3	15.7%
**2004**	10.2	36.4%	2,617.6	14.0%
**2005**	29.2	33.5%	3,481.5	16.6%
**2006**	17.1	25.1%	3,859.7	16.9%
**2007**	16.1	25.2%	4,373.3	16.7%
**2008**	12.3	26.9%	5,715.2	18.6%
**2009**	16.1	25.8%	5,417.6	17.4%
**2010**	25.0	32.5%	5,810.9	16.7%
**2011**	24.0	31.4%	6,192.6	17.0%
**2012**	26.5	33.7%	6,274.0	17.0%
**2013**	39.5	36.2%	6,757.7	16.8%
**2014**	39.9	37.0%	6,233.9	16.9%
**2015**	51.7	45.0%	6,681.9	18.5%
**Total**	364.1	32.5%	72,439.5	17.0%

DAH, development assistance for health; DAMH, development assistance for mental health.

Appendix 3. Annual philanthropic DAH for mental health and other health conditions between 2000 and 2015 (million, 2017 US$)

	2000	2001	2002	2003	2004	2005	2006	2007	2008	2009	2010	2011	2012	2013	2014	2015	Total
**Mental Health**	20.0	18.8	10.4	7.4	10.2	29.2	17.1	16.1	12.3	16.1	25.0	24.0	26.5	39.5	39.9	51.7	364.1
HIV/AIDS	213.7	246.4	427.9	447.7	528.3	741.1	942.5	1,074.8	1,389.5	1,104.0	1,174.6	1,027.8	996.1	671.4	671.3	621.8	12,278.9
Tuberculosis	28.8	34.8	42.4	56.6	75.6	68.4	145.0	216.5	306.1	268.0	307.9	279.7	287.6	236.0	201.0	240.4	2,794.8
Malaria	51.1	44.0	22.8	43.2	82.5	124.6	132.8	219.8	310.4	249.3	245.2	210.3	199.4	185.3	153.0	178.2	2,451.9
Other Infectious Diseases	141.8	134.5	97.5	108.0	113.0	122.7	128.8	176.5	283.7	249.7	252.8	291.1	271.5	289.9	398.3	356.9	3,416.8
Maternal Health	386.7	390.0	278.7	296.8	256.6	309.5	336.5	399.6	415.3	520.8	509.6	599.9	549.4	497.2	503.9	543.2	6,793.8
Newborn and Child Health	591.9	680.3	543.3	856.4	700.6	976.0	890.4	1,086.7	1,272.8	1,287.5	1,513.4	1,739.1	1,964.6	2,156.3	1,909.1	2,241.5	20,409.9
Non-communicable Diseases (excl mental health)	56.8	67.8	70.3	83.7	80.1	82.7	118.3	128.7	183.6	186.9	214.9	183.5	173.8	233.9	240.0	276.7	2,381.6
Health Sector Programme Support	186.8	205.4	176.6	214.1	212.1	254.9	232.8	231.1	441.4	484.3	557.4	544.6	541.9	752.0	676.0	735.1	6,446.5
Other Health Focus Areas	376.0	410.9	478.8	474.5	558.7	772.3	915.5	823.6	1,100.1	1,050.9	1,010.1	1,292.6	1,263.3	1,696.2	1,441.3	1,436.3	15,101.1
Total	2,053.6	2,232.9	2,148.7	2,588.3	2,617.6	3,481.5	3,859.7	4,373.3	5,715.2	5,417.6	5,810.9	6,192.6	6,274.0	6,757.7	6,233.9	6,681.9	72,439.5

DAH, development assistance for health; DAMH, development assistance for mental health; HIV/AIDS, human immunodeficiency virus infection and acquired immune deficiency syndrome.

Appendix 4. Annual philanthropic DAMH and annual philanthropic DAH between 2000 and 2015, by channel organisation (million, 2017 US$)

	2000	2001	2002	2003	2004	2005	2006	2007	2008	2009	2010	2011	2012	2013	2014	2015	Total
**Philanthropic DAMH**																	
Bilateral GOs	0.0	0.0	0.0	0.0	0.0	0.0	0.0	0.0	0.0	0.0	0.0	0.0	0.0	0.0	0.0	0.0	0.0
Multilateral GOs	5.9	5.8	1.1	1.1	0.8	0.7	0.9	0.9	0.0	0.0	0.0	0.0	3.0	3.3	3.0	4.5	30.9
Multilateral DFIs	0.0	0.0	0.0	0.0	0.0	0.0	0.0	0.0	0.0	0.0	0.0	0.0	0.0	0.0	0.0	0.0	0.0
US Foundations	6.5	4.8	5.8	3.3	4.9	2.6	2.1	1.9	3.0	4.0	3.8	3.0	9.0	6.1	11.2	7.2	79.3
NGOs	7.6	8.2	3.5	3.0	4.5	25.9	14.1	13.2	9.3	12.1	21.2	21.0	14.5	30.1	25.8	40.0	254.0
GHIs	0.0	0.0	0.0	0.0	0.0	0.0	0.0	0.0	0.0	0.0	0.0	0.0	0.0	0.0	0.0	0.0	0.0
Sub-Total	20.0	18.8	10.4	7.4	10.2	29.2	17.1	16.1	12.3	16.1	25.0	24.0	26.5	39.5	39.9	51.7	364.1
**Philanthropic DAH**																	
Bilateral GOs	0.0	0.0	0.0	0.0	0.0	0.0	0.0	0.0	0.0	0.0	0.0	0.0	0.0	0.0	0.0	0.0	0.0
Multilateral GOs	317.6	364.9	272.8	269.0	339.8	413.1	423.4	477.8	624.3	628.9	847.8	920.8	675.4	729.6	675.3	920.9	8,901.3
Multilateral DFIs	0.0	0.0	0.0	0.0	0.0	0.0	0.0	0.0	0.0	0.0	0.0	0.0	0.0	0.0	0.0	0.0	0.0
US Foundations	624.7	541.0	589.6	847.0	563.5	734.1	1,008.9	1,257.7	1,743.8	1,679.9	1,521.7	1,698.8	1,771.9	1,826.4	1,888.2	2,059.6	20,356.8
NGOs	1,107.9	1,182.4	1,284.9	1,449.1	1,678.7	2,200.1	2,341.5	2,401.8	3,099.5	2,887.1	3,201.4	3,147.0	3,381.0	3,666.2	3,240.5	3,065.2	39,334.2
GHIs	3.4	144.6	1.4	23.2	35.7	134.1	86.0	236.0	247.6	221.6	239.9	426.0	445.8	535.6	429.9	636.2	3,847.2
Sub-Total	2,053.6	2,232.9	2,148.7	2,588.3	2,617.6	3,481.5	3,859.7	4,373.3	5,715.2	5,417.6	5,810.9	6,192.6	6,274.0	6,757.7	6,233.9	6,681.9	72,439.5

DAH, development assistance for health; DAMH, development assistance for mental health; DFIs, development finance institutions; GHIs, global health initiatives; GOs, governmental organisations; NGOs, non-governmental organisations; US, United States.

Appendix 5. Cumulative philanthropic DAMH by the top 10 US foundations as channels between 2000 and 2015, by region and country income group (thousand, 2017 US$)

	Philanthropic DAMH (US$, thousand)
**Income Group**	
LIC	
Ford Foundation	1,736
David and Lucile Packard Foundation	727
Rockefeller Foundation	447
Open Society Fund	425
Foundation to Promote Open Society	257
Draper Richards Kaplan Foundation	236
Michael and Susan Dell Foundation	165
Bristol-Myers Squibb Foundation, Inc	143
Mulago Foundation	123
James S. McDonnell Foundation	115
LMC	
Ford Foundation	5,243
Partridge Foundation	2,011
Open Society Fund	1,418
W. K. Kellogg Foundation	1,243
Foundation to Promote Open Society	1,063
China Medical Board, Inc	948
James S. McDonnell Foundation	385
Eli Lilly and Company Foundation	344
Sorenson Legacy Foundation	310
Abbott Fund	292
UMC	
James S. McDonnell Foundation	2,913
Foundation to Promote Open Society	2,100
Open Society Fund	1,834
Ford Foundation	990
Greater Houston Community Foundation	927
China Medical Board, Inc	921
MetLife Foundation	920
Alcoa Foundation	470
Harold K. L. Castle Foundation	223
Paso del Norte Health Foundation	207
**WHO Region**	
AFR	
Ford Foundation	1,325
Rockefeller Foundation	371
David and Lucile Packard Foundation	350
Draper Richards Kaplan Foundation	306
Michael and Susan Dell Foundation	201
Open Society Fund	193
Mulago Foundation	161
Oprah Winfrey Foundation	134
Bristol-Myers Squibb Foundation, Inc	124

	Philanthropic DAMH (US$, thousand)
William and Flora Hewlett Foundation	115
AMR	
James S. McDonnell Foundation	2,978
Foundation to Promote Open Society	2,452
Open Society Fund	2,020
W. K. Kellogg Foundation	1,435
MetLife Foundation	920
Alcoa Foundation	482
Harold K. L. Castle Foundation	223
Paso del Norte Health Foundation	207
Ford Foundation	195
Dalio Foundation, Inc	180
EMR	
Ford Foundation	666
Foundation to Promote Open Society	573
Sorenson Legacy Foundation	506
David and Lucile Packard Foundation	416
Open Society Fund	269
Eli Lilly and Company Foundation	241
Robert Wood Johnson Foundation	112
Alphawood Foundation	104
Draper Richards Kaplan Foundation	83
Johnson & Johnson Family of Companies Foundation	74
EUR	
Open Society Fund	862
James S. McDonnell Foundation	449
Pfizer Foundation, Inc	360
Bristol-Myers Squibb Foundation, Inc	347
Foundation to Promote Open Society	211
Medtronic Foundation	189
Ford Foundation	156
John D. and Catherine T. Macarthur Foundation	120
Eli Lilly and Company Foundation	112
Charles Stewart Mott Foundation	110
SEAR	
Ford Foundation	671
Open Society Fund	291
Abbott Fund	249
Nike Foundation	235
Robert Wood Johnson Foundation	160
David and Lucile Packard Foundation	156
Foundation to Promote Open Society	144
Annie E. Casey Foundation	116
Rockefeller Foundation	79
Alphawood Foundation	42
WPR	
Ford Foundation	4,956
Partridge Foundation	2,011
China Medical Board, Inc	1,869
Greater Houston Community Foundation	927
Bloomberg Philanthropies	173

	Philanthropic DAMH (US$, thousand)
UPS Foundation	157
Starr Foundation	141
Timken Foundation of Canton	84
Johnson & Johnson Family of Companies Foundation	68
Rockefeller Foundation	63
**Multiple Regions**	
Simons Foundation	7,087
Oak Foundation U.S.A.	5,554
Conrad N. Hilton Foundation	3,560
Foundation to Promote Open Society	2,088
Open Society Fund	1,929
Carmel Hill Fund	1,864
Skoll Foundation	1,532
Silicon Valley Community Foundation	1,481
Eli Lilly and Company Foundation	1,216
MetLife Foundation	1,088
Robert Wood Johnson Foundation	826
Stewardship Foundation	715
**Unallocated/Unspecified**	
Ford Foundation	2,525
James S. McDonnell Foundation	1,697
Bill & Melinda Gates Foundation	1,513
Rockefeller Brothers Fund, Inc	468
Alcoa Foundation	407
John D. and Catherine T. Macarthur Foundation	391
Draper Richards Kaplan Foundation	334
Schmidt Family Foundation	279
Pfizer Foundation, Inc	224
Sall Family Foundation, Inc	128
Helen Bader Foundation, Inc	112
State Street Foundation	105

The table reports philanthropic DAMH disbursed by US foundations as channels (i.e. intermediary organisations disbursing funding to implementing institutions providing support in low- and middle-income countries). It is worth noting that a much larger amount could have been disbursed by US foundations through other channels (e.g. non-governmental organisations and United Nations agencies). DAMH, development assistance for mental health; AFR, African region; AMR, region of the Americas; EMR, Eastern Mediterranean region; EUR, European Region; SEAR, South-East Asia region; WPR, Western Pacific region; LIC, low-income countries; LMC, lower middle-income countries; UMC, upper middle-income countries.

Appendix 6. Cumulative philanthropic DAMH and philanthropic DAH between 2000 and 2015, by region, country income group, country (thousand, 2017 US$)

	Philanthropic DAMH		Philanthropic DAH	
	US$ (thousand)	% Total DAMH	US$ (thousand)	% Total DAH
**Total**	364,134.6	32.5%	72,439,528.0	17.0%
**Income Group**				
LIC	5,485.4	5.0%	5,132,183.0	4.4%
LMC	15,659.7	12.3%	4,498,688.5	5.6%
UMC	13,998.3	25.8%	1,318,289.9	3.7%
HIC	38.4	78.5%	1,768.2	17.6%
**WHO Region**				
AFR	3,867.3	4.7%	5,718,036.5	4.6%
AMR	11,807.0	33.7%	1,023,675.8	3.4%
EMR	3,226.9	3.7%	927,386.6	4.5%
EUR	3,095.4	7.7%	311,805.8	2.9%
SEAR	2,305.0	11.6%	2,118,801.3	7.5%
WPR	10,880.3	42.5%	851,223.3	4.7%
**Country**				
Afghanistan	408.7	1.7%	94,195.7	2.7%
Albania	189.1	4.9%	11,487.3	3.2%
Algeria	6.7	1.1%	1,086.4	1.8%
Angola	4.5	1.0%	66,392.0	4.5%
Antigua and Barbuda	188.1	98.5%	313.0	6.6%
Argentina	348.4	68.1%	34,063.2	0.9%
Armenia	1.3	0.2%	6,993.8	1.8%
Azerbaijan	1.1	0.2%	10,192.5	3.3%
Bahrain	0.0	−	0.0	0.0%
Bangladesh	14.6	1.0%	317,378.9	6.9%
Barbados	0.0	−	5.8	0.0%
Belarus	2.7	3.6%	8,260.7	5.3%
Belize	99.2	74.2%	3,681.8	6.0%
Benin	2.0	0.9%	53,032.8	3.9%
Bhutan	0.1	0.9%	3,699.9	4.8%
Bolivia	408.8	10.3%	45,243.5	3.5%
Bosnia and Herzegovina	813.1	5.6%	10,098.0	2.2%
Botswana	0.0	−	92,161.1	5.5%
Brazil	1,854.1	34.5%	108,092.7	3.2%
Bulgaria	0.0	−	4,838.5	1.1%
Burkina Faso	2.6	1.4%	99,125.9	5.4%
Burundi	38.5	1.0%	48,216.0	4.3%
Cambodia	46.1	0.9%	97,067.5	4.1%
Cameroon	177.7	26.2%	89,223.5	6.5%
Cape Verde	0.0	−	930.7	0.5%
Central African Republic	3.6	0.9%	18,448.4	5.5%
Chad	13.7	2.9%	34,505.7	4.9%
Chile	1,573.5	87.6%	9,716.1	8.6%

	Philanthropic DAMH		Philanthropic DAH	
	US$ (thousand)	% Total DAMH	US$ (thousand)	% Total DAH
China	6,329.3	80.1%	411,860.8	9.6%
Colombia	664.8	47.2%	82,663.2	2.3%
Comoros	0.0	–	2,908.3	3.0%
Congo (Brazzaville)	0.6	10.5%	13,023.5	5.4%
Costa Rica	113.0	62.7%	4,269.2	3.2%
Cote d'Ivoire	28.1	1.5%	71,733.1	3.7%
Croatia	0.0	0.0%	268.0	0.3%
Cuba	41.1	5.3%	10,840.0	4.7%
Czech Republic	0.0	–	320.1	100.0%
Democratic Republic of the Congo	221.5	1.6%	290,804.2	5.2%
Djibouti	0.0	–	5,482.3	3.0%
Dominica	0.0	–	327.9	4.1%
Dominican Republic	59.7	8.2%	29,296.5	2.0%
Ecuador	197.8	29.6%	38,981.8	5.9%
Egypt	354.1	9.6%	44,569.3	3.0%
El Salvador	877.3	95.5%	19,484.9	3.0%
Equatorial Guinea	0.0	–	3,085.5	5.3%
Eritrea	2.1	0.9%	22,054.6	4.1%
Estonia	0.0	–	202.6	2.7%
Ethiopia	287.3	14.5%	617,481.4	6.1%
Federated States of Micronesia	0.0	–	1,600.8	0.6%
Fiji	0.0	0.0%	2,554.7	1.5%
Gabon	0.0	–	7,727.8	6.0%
Georgia	7.1	0.2%	10,760.8	1.9%
Ghana	53.5	2.3%	224,224.5	5.6%
Grenada	232.9	98.6%	832.9	8.2%
Guatemala	127.2	78.0%	48,866.5	3.2%
Guinea	0.0	10.5%	48,181.0	4.9%
Guinea-Bissau	2.5	0.9%	11,619.4	3.7%
Guyana	11.3	4.9%	9,187.1	2.4%
Haiti	256.9	29.4%	106,615.6	3.5%
Honduras	448.3	12.7%	38,122.2	3.5%
Hungary	243.4	100.0%	4,296.7	56.0%
India	1,257.0	24.4%	1,368,016.4	10.2%
Indonesia	485.6	27.4%	163,044.1	3.3%
Iran	212.3	86.2%	8,573.0	3.4%
Iraq	6.1	0.8%	8,440.9	0.5%
Jamaica	51.9	74.2%	12,333.0	3.2%
Jordan	284.3	13.2%	10,883.1	1.2%
Kazakhstan	30.9	11.5%	9,494.2	2.0%
Kenya	1,300.6	34.9%	434,123.8	4.2%
Kiribati	0.0	0.0%	659.7	0.9%
Kosovo	54.0	1.7%	3,725.1	3.0%
Kyrgyzstan	62.9	28.3%	13,873.4	2.1%
Laos	30.1	9.8%	25,829.2	3.1%
Latvia	0.0	–	114.0	0.1%
Lebanon	31.7	0.7%	5,489.2	2.4%
Lesotho	21.3	92.3%	26,566.8	2.9%
Liberia	137.5	7.4%	92,861.2	6.9%

	Philanthropic DAMH		Philanthropic DAH	
	US$ (thousand)	% Total DAMH	US$ (thousand)	% Total DAH
Libya	4.9	0.7%	667.9	0.7%
Lithuania	0.0	–	78.9	0.3%
Macedonia	2.8	1.3%	3,989.3	2.4%
Madagascar	17.6	0.9%	74,886.7	4.6%
Malawi	125.2	1.3%	199,058.0	4.3%
Malaysia	80.6	43.2%	5,975.8	9.3%
Maldives	0.0	–	406.9	2.4%
Mali	51.8	8.5%	108,199.0	4.7%
Marshall Islands	0.0	–	1,627.5	1.3%
Mauritania	0.0	–	10,708.2	4.0%
Mauritius	0.0	–	986.7	5.7%
Mexico	3,361.6	90.0%	214,112.6	6.3%
Moldova	668.8	8.9%	20,095.9	4.0%
Mongolia	5.2	0.4%	6,861.6	1.8%
Montenegro	8.6	2.8%	1,235.0	2.0%
Morocco	315.3	35.6%	22,383.1	1.4%
Mozambique	55.8	1.3%	171,316.4	2.3%
Myanmar	224.1	87.4%	89,266.4	6.8%
Namibia	18.8	11.3%	28,034.6	1.8%
Nepal	31.6	1.0%	67,265.7	3.6%
Nicaragua	115.8	15.6%	40,251.0	3.0%
Niger	10.1	1.1%	62,211.8	5.5%
Nigeria	312.2	17.8%	892,400.7	7.2%
North Korea	0.3	0.4%	14,018.2	8.3%
Oman	0.0	–	0.0	0.0%
Pakistan	632.4	48.4%	501,260.1	8.7%
Palau	0.0	–	199.8	1.1%
Palestine	813.0	2.0%	12,146.5	1.2%
Panama	10.2	29.8%	5,176.1	2.1%
Papua New Guinea	0.0	–	34,662.1	2.0%
Paraguay	16.1	3.7%	10,540.4	3.4%
Peru	513.6	11.7%	139,581.8	7.5%
Philippines	3,744.5	84.6%	68,001.1	2.5%
Poland	228.4	100.0%	4,045.4	8.7%
Romania	202.8	100.0%	11,421.2	1.2%
Russia	336.6	100.0%	38,840.5	4.9%
Rwanda	103.7	1.0%	165,849.8	4.3%
Saint Kitts and Nevis	0.0	–	141.7	2.0%
Saint Lucia	22.0	4.3%	1,009.2	1.7%
Saint Vincent and the Grenadines	164.8	6.0%	593.9	3.2%
Samoa	0.0	–	349.6	0.3%
Sao Tome and Principe	0.0	–	3,480.1	3.2%
Saudi Arabia	0.0	–	1.1	0.2%
Senegal	6.6	1.0%	91,509.5	4.1%
Serbia	73.4	4.3%	9,314.6	2.3%
Seychelles	0.0	–	363.2	3.4%
Sierra Leone	25.4	0.9%	70,217.8	6.6%
Slovakia	43.3	100.0%	161.4	0.4%
Solomon Is.	0.0	0.0%	3,756.6	1.1%

	Philanthropic DAMH		Philanthropic DAH	
	US$ (thousand)	% Total DAMH	US$ (thousand)	% Total DAH
Somalia	4.6	1.0%	27,953.5	4.8%
South Africa	373.4	6.3%	344,333.3	3.9%
South Korea	0.0	–	51.7	100.0%
South Sudan	0.1	10.5%	39,223.6	3.0%
Sri Lanka	81.9	1.2%	16,421.1	2.8%
Sudan	30.1	4.0%	113,264.6	8.3%
Suriname	25.4	99.2%	4,924.3	2.8%
Swaziland	0.1	0.9%	30,218.2	4.5%
Syria	69.5	1.0%	11,679.8	6.8%
Tajikistan	19.9	35.2%	17,941.1	3.0%
Tanzania	221.3	5.4%	364,214.2	3.5%
Thailand	190.9	27.6%	72,798.2	6.8%
The Gambia	0.1	2.5%	40,551.2	10.4%
Timor Leste	18.9	3.9%	6,485.4	2.4%
Togo	8.8	0.9%	27,858.2	6.3%
Tonga	0.0	–	347.6	0.3%
Trinidad and Tobago	0.0	–	20.3	0.0%
Tunisia	59.8	16.3%	4,091.6	1.7%
Turkey	47.7	3.9%	28,336.9	2.6%
Turkmenistan	0.0	–	2,528.5	1.8%
Uganda	68.1	3.0%	275,958.9	3.4%
Ukraine	57.2	14.3%	53,426.4	5.0%
Uruguay	17.4	3.9%	1,029.4	0.5%
Uzbekistan	0.2	0.2%	25,464.9	3.4%
Vanuatu	0.0	0.0%	3,254.7	1.8%
Venezuela	6.0	3.5%	3,358.5	2.8%
Vietnam	644.3	20.4%	186,562.1	4.5%
Yemen	0.1	8.2%	56,305.0	4.9%
Zambia	26.0	1.2%	215,219.6	3.4%
Zimbabwe	137.8	16.1%	131,719.4	4.0%
**Multiple Regions**	35,010.1	66.4%	4,700,082.5	8.0%
**Unallocated/ Unspecified**	293,942.7	37.8%	56,788,516.0	41.6%

DAH, development assistance for health; DAMH, development assistance for mental health; AFR, African region; AMR, region of the Americas; EMR, Eastern Mediterranean region; EUR, European region; SEAR, South-East Asia region; WPR, Western Pacific region; LIC, low-income countries; LMC, lower middle-income countries; UMC, upper middle-income countries.
